# Language Representation Models: An Overview

**DOI:** 10.3390/e23111422

**Published:** 2021-10-28

**Authors:** Thorben Schomacker, Marina Tropmann-Frick

**Affiliations:** Department of Computer Science, Hamburg University of Applied Sciences, 20099 Hamburg, Germany; thorben.schomacker@haw-hamburg.de

**Keywords:** natural language processing, neural networks, transformer, embeddings, multi-task learning, attention-based models, deep learning

## Abstract

In the last few decades, text mining has been used to extract knowledge from free texts. Applying neural networks and deep learning to natural language processing (NLP) tasks has led to many accomplishments for real-world language problems over the years. The developments of the last five years have resulted in techniques that have allowed for the practical application of transfer learning in NLP. The advances in the field have been substantial, and the milestone of outperforming human baseline performance based on the general language understanding evaluation has been achieved. This paper implements a targeted literature review to outline, describe, explain, and put into context the crucial techniques that helped achieve this milestone. The research presented here is a targeted review of neural language models that present vital steps towards a general language representation model.

## 1. Introduction

Text mining, as a subset of data mining, is the process in which algorithms try to discover and extract knowledge from a text that has no apparent structure. Text mining thus deals with information retrieval (e.g., documents or websites), classification (e.g., news tagging), clustering, and other similar tasks. However, text mining has a limit when it comes to “understanding” the mined results.

The discipline of natural language processing (NLP) tries to understand and exploit natural language. It covers a wide range of tasks such as translation, summarizing, or question-answering. Thus, text mining and NLP are closely related and intertwined approaches to text processing. Researchers and practitioners of NLP use tools available from linguistic toolboxes, such as the part of speech, which denotes how a given word functions within a sentence in its two essences: the meaning and the structure (grammar). Grammatical structures are presented as phrases or dependency relations. NLP tackles the problems of anaphora (an expression that depends on an antecedent expression), cataphora (the use of an expression that depends on a postcedent expression), and ambiguities of words and of grammatical structures (what a given word or prepositional phrase is modifying). In dealing with these problems, the NLP uses knowledge representations (such as word lexicons with word meanings, grammatical properties, grammar rules, a thesaurus of synonyms, a list of abbreviations, and many kinds of ontologies).

Applying neural networks and deep learning techniques to NLP led to a new way of solving NLP tasks: *neural language models*. These models are very successful in solving numerous NLP tasks. The introduction of transfer learning to NLP has brought a major breakthrough in NLP: machines have exceeded human performance in several NLP tasks. The progress of natural language models is being actively monitored and assessed by the open General Language Understanding Evaluation (GLUE) benchmark score platform [1] (https://gluebenchmark.com, accessed on 2 January 2021). The goal of this paper is to provide readers with an overview of the most-used concepts and milestones of the last five years in the area of NLP. This paper aims to explain the most important concepts behind the language representation models that have helped achieve the milestone, i.e., develop the models to outperform the human baseline on the GLUE benchmark (https://gluebenchmark.com/leaderboard, accessed on 2 January 2021). The research question is as follows: which concepts and milestones in the area of natural language processing are the most important ones to help outperform the human baseline performance.

## 2. Related Works

Language representation and neural language models are rapidly growing fields that will probably play a substantial role in the future of NLP. A few studies have conducted surveys of the field.

For instance, ref. [2] conducted a short survey of neural language models and possible evaluation tasks and corpora.

Another example is [3]; in this work, a pre-trained neural language model taxonomy was conducted.

Similarly, ref. [4] provided a systemization of many language representation models by representation level, input level, model type, model architecture, and model supervision.

All of these three surveys lack information about novel techniques and models that contributed to the breakthrough of surpassing the baseline human performance.

The authors of [5] conducted a rich survey of language representation models, including many models from 2020 and 2021. They additionally provided useful insights about pre-processing, classification techniques, evaluation metrics, and applications. Again, the survey neither focused on breakthrough methods and techniques nor on neural language models.

In contrast to these related works, our work focuses on language representation models that outperform the human benchmark on the GLUE score. It explains the concepts and background of these models in an articulated and systematic way.

## 3. Targeted Literature Review

The scope of this targeted review about neural language models is papers describing the concepts, internals, and descriptions of these models and is evaluated using the GLUE benchmark [1]. There are 77 models listed on the benchmark’s leaderboard. We discarded the models that do not outperform human baselines (ranked 16/77). Of the 15 remaining models, we review the models developed in the last five years, i.e., since 2016, starting with the attention-based concepts [6] and the transformer architecture in 2017 [7] and ending with DeBERTa in 2021 [8]. The reason for limiting the targeted review to the last 5 years is to show the quick progress achieved recently. The concepts and milestones of the latest research in natural language models are described in this paper.

**Figure 1:**
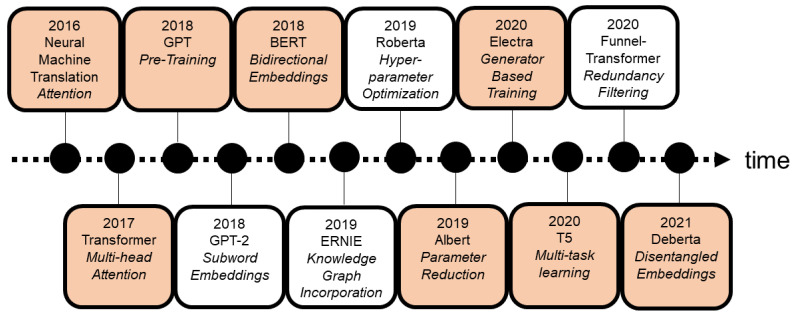
Selected overview of the milestones in neural language models of the last 5 years. Chronologically ordered.

Figure 1 depicts a few of these models and their novel techniques or concepts. Additionally, it shows how rapid new milestones and novel techniques have been published in the last years. Table 1 gives an overview of a model’s GLUE benchmark score, the parameters, and the number of steps of each of the models discussed in this paper. The models and concepts in the orange highlighted boxes are further discussed in this paper. The concepts of Roberta (hyper-parameter optimization, 2019) and Funnel (transformer redundancy filtering, 2020) are not covered in detail because they have not introduced a substantial improvement over the initial proposal, as is also the case with GPT2 (subword embeddings, 2018) and ERNIE (knowledge graph incorporation, 2019). Table 1 allows the reader to obtain a general overview of the particular performance and efficiency of the key models.

**Figure 2:**
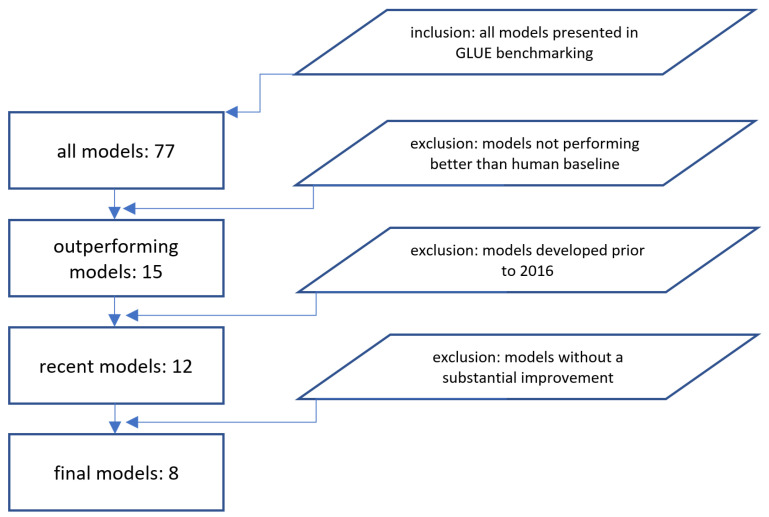
Inclusion and exclusion criteria.

Figure 2 presents the inclusion/exclusion criteria of models in our overview.

## 4. Key Concepts of Neural Language Models

In this section, we present key concepts of natural language models and how they connect, and how each of the concepts was further developed and refined by the next one. First, we introduce the basic and starting concept of word representation in Section 4.1. Built on this topic, Section 4.2 talks about sequence-to-sequence models and how they process sequential information (e.g., texts). Section 4.3 describes how encoder-decoder models boost the capabilities of sequence-to-sequence models. Next, Section 4.4 shows how attention helps encoder-decoder models to better understand longer sequences. Section 4.5 shows how transfer learning becomes applicable in NLP and how it helps developing better performing models. Section 4.6 explains how a bidirectional, thereby bigger, context scope for word representations helps models to gain a better understanding of their input words and phrases. This bidirectionality becomes possible by using masked language modelling (MLM). Section 4.7 shows how further refining these word representations can significantly increase the model’s performance. Section 4.8 illustrates how to improve the model’s performance and efficiency by expanding the MLM task into a generator and a discriminator. Next, Section 4.9 showcases the positive effect of a few parameter reduction techniques and how they help in building more efficient models. Additionally, Section 4.10 explores the current possibilities and results of multi-task trained models. Section 4.11 delivers a recent use-case of applying transformer models to the machine translation. In Section 6, we discuss recent milestones and provide the reader with an outlook on what areas could become important in the future of neural language models.

### 4.1. Word Representations

**Figure 3:**
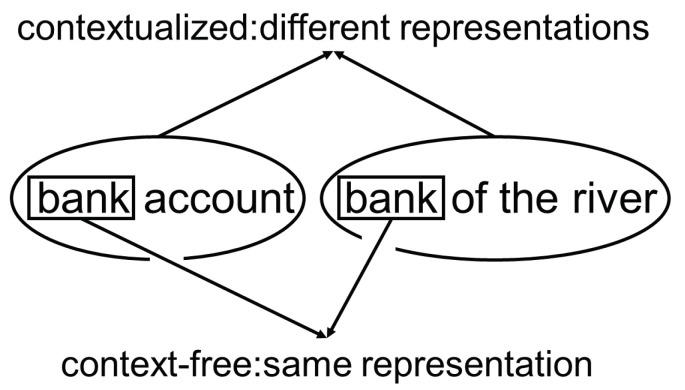
Example comparison of the representations of the same span of characters in a contextualized and in a not contextualized model.

The basis for each neural language model is vocabulary. A vocabulary is a table that assigns each token or word a representation. This representation could be a number or a multi-dimensional vector. These representations are usually called *word embeddings*. There are a few different implementations of word embeddings. They can be categorized into *context-free* and *contextualized* word embeddings. Context-free representations such as Glove [14] and Word2Vec [15] are based only on the characters the word consists of. Contextualized representations are based on the character span of the word and enhancing the representation by the words surrounding the word. Figure 3 illustrates an example that shows the different representations of the word “bank”. In the left sentence, it means a credit institution, and in the right sentence, a geographical phenomenon, although it has the same span of characters.

### 4.2. Sequence-To-Sequence Models

**Figure 4:**
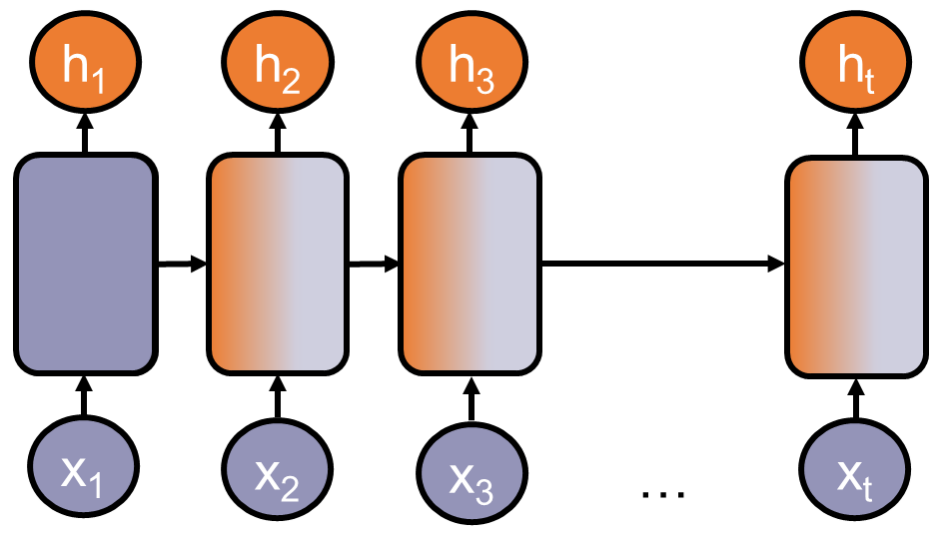
Schematic depiction of a Recurrent Neural Network (RNN) (modified from [18]).

Sequence-to-sequence models are specialized in processing a sequential input, e.g., a list of numbers or a consequential sentence, and producing a sequential output. Probably the most used architecture families are recurrent neural networks or *RNNs*, introduced by [16]. They are well applicable to language tasks because many tasks rely on sequential data such as continuous texts. A key strength of most RNNs is that they can process and generalize across sequences of values of variable lengths. This strength relies on the model’s ability to share parameters across different parts of a model. Thereby, RNNs can connect information that is distributed across the sequence: for example, where the model’s task is to extract temporal information from a sequence. It has the following two sentences as input: “Today I am feeling sick” and “I am feeling sick today”. In both cases, it should extract “today” independently from its position in the sequence. Ref. [17], p. 373, describes RNNs as a chain of multiple copies of the same network, each one sharing the parameters with its succeeding copy. Figure 4 depicts a chained neural network.

### 4.3. Encoder-Decoder Models

**Figure 5:**
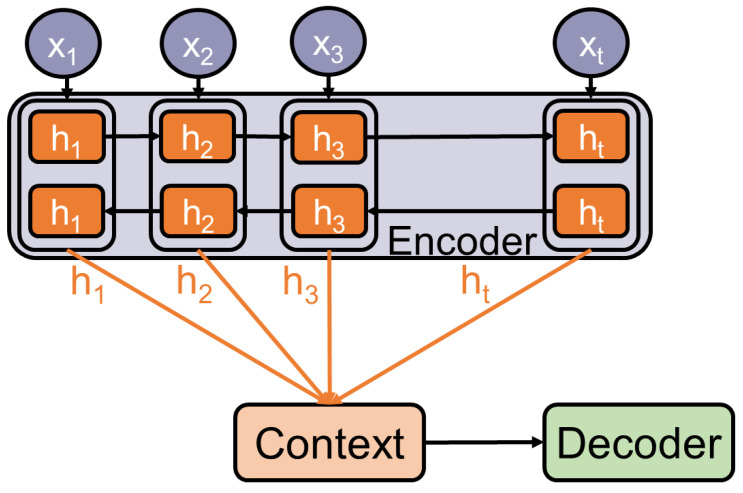
Illustration of the Encoder-Decoder Architecture.

The major limitation of the previously discussed RNNs is that their output length is determined by the input length. Many tasks such as translation depend on this feature. The first achievements to overcome this limitation are [19,20]. They introduce the encoder-decoder framework and apply it to the task of neural machine translation. These frameworks consist of three main components as they are depicted in Figure 5:

1. *Encoder:* Extracts features by reading and converting the input into distributed representations, with one feature vector associated with each word position.
2. *Context:* Either a feature vector or a list of feature vectors based on the extracted features. If it is a list of feature vectors, it has the benefit that each feature vector can be accessed independently of its position in the input.
3. *Decoder:* Sequentially processes the context to generate the final output and solve the task.

### 4.4. Attention

**Figure 6:**
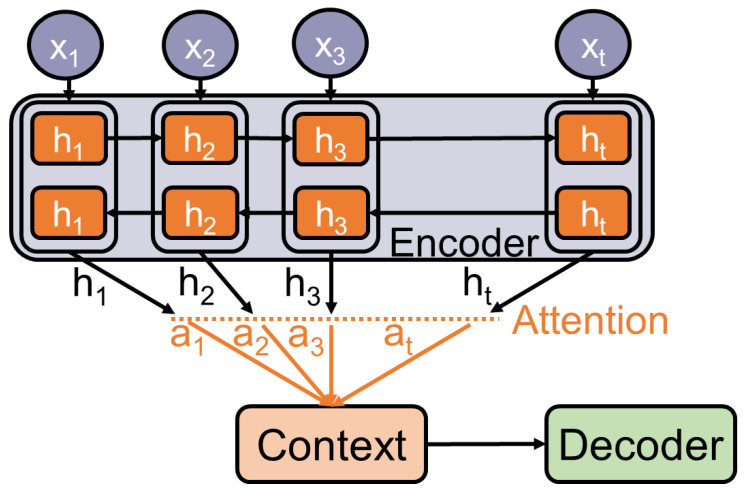
Visualization of the attention mechanism applied to an encoder-decoder model.

**Figure 7:**
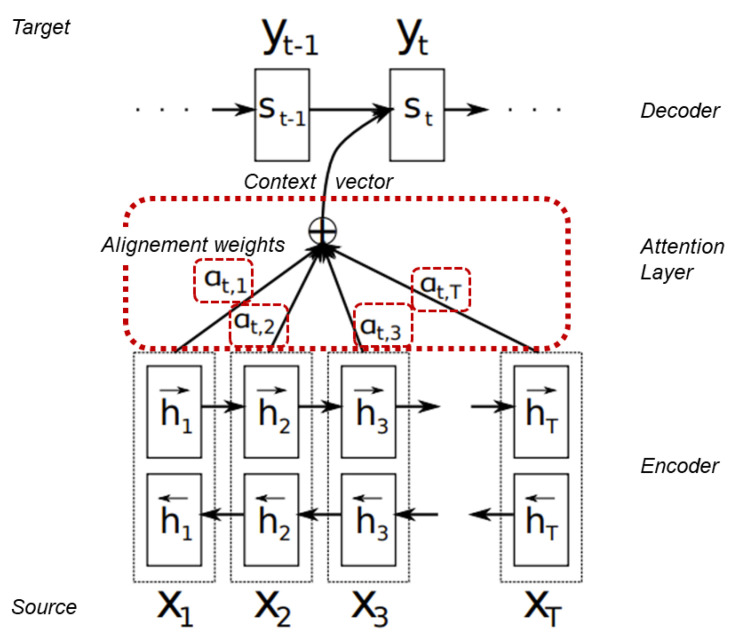
Visualization of Additive Attention mechanism. Modified from [6]. The emphasized text gives further information about parts of the process.

The encoder-decoder framework performs efficiently on a variety of tasks, e.g., machine translation [21]. However, the fact that all the information is stored in a context can limit the framework’s ability to understand long and complex inputs. One approach to overcome this limitation is the *attention mechanism*. It was firstly applied to machine translation by [6,22]. The main idea is similar to the way humans perceive and understand their environment. Not all information has the same influence on the output. This mechanism is built on the encoder-decoder idea but differs from it in two main ways. Firstly, the encoder passes allthe hidden states to the decoder. Secondly, an additional attention step is performed by the decoder, in which each annotation receives a weight that further determines its amount of influence on the output. The box, with the dashed outline, in Figure 6 illustrates the attention step in additive attention, the kind of attention [6] (see Figure 7). used. There are different kinds of attention, which all share the core idea of calculating and assigning weights to annotations.

**Figure 8:**
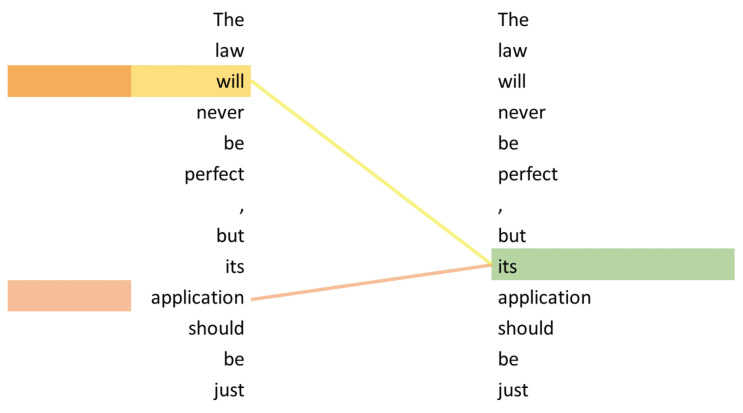
Example of how self-attention influences the representation of the word “its” (modified from [7]).

Currently, probably the most used application of the attention mechanism is the *transformer* architecture, which was introduced by [7]. It uses *self-attention*, which is an extension of the attention mechanism. The main advantage of self-attention is its auto-regressive property. This means that the model is able to look back to previously processed inputs while encoding the current one. The example in Figure 8 illustrates self-attention. The word “it’s” is correctly associated with the “law”, allowing the model to possibly understand that “it” is semantically equivalent to “the law”. Ref. [23] describes the process of self-attention by dividing it into six steps. In step one, each encoder input word (represented by a vector) is multiplied by three weight matrices, resulting in three different vectors: a query vector, a key vector, and a value vector for each word. These weight matrices are trained in the training process. In step two, the self-attention is calculated for each word. This is done by individually calculating the dot-product of the word’s query vector $q_{i}$ and the key vector of each word ($k1_{1},\cdots ,k_{n}$) in the sentence, resulting in *n* scores. In steps three and four, the scores are divided by the square root (e.g., 8 in [7]) of the number of dimensions (e.g., 64 in [7]). Afterward, these scores are all normalized using Softmax. These scores indicate how much each word is expressed at the current position. In step five, each value vector is multiplied by the Softmax score. In the final step, the weighted value vectors are added together and form the output of the self-attention layer of this position.

In addition to the content of the word or phrase, ref. [7] injected positional information into the embedding. This positional information is important to enable the model to make use of the order of the input sequence. Their positional encoding represents the absolute position of the word or phrase in the sequence. Later papers discussed the usage of relative position embeddings (e.g., [24]). The results of [25] showed that relative position representations are more effective for natural language understanding and generation tasks. This injection of language information is a core feature of so-called *language-representation models*, models that are specialized in representing valuable natural language information. Another feature of the transformer by [7] is sharing parameters across layers. With *parameter sharing*, the overall number of parameters a model should learn can be significantly reduced.

### 4.5. Transfer Learning and Pre-Trained Models

Textual data exist abundantly. However, task-specific and labelled data are sparse and expensive to acquire. One way to boost the model’s performance, when only few task-specific data are available, is *pre-training*. The approach introduced by [26] used the transformer architecture in their pre-training setup. They first pre-trained the model in an unsupervised way on a large data set to gain a general or world understanding. Then, the model’s weights are used as the foundation of the semi-supervised fine-tuning on the task-specific data to solve the task. This approach is called *transfer learning*. Human intelligence is very capable of it. By writing many essays in school, humans not only become better at the concrete task but are able to generalize information and capabilities: for example, applying the knowledge to other similar tasks such as writing a business e-mail or a letter of application.

### 4.6. Bidirectional Representations

**Figure 9:**
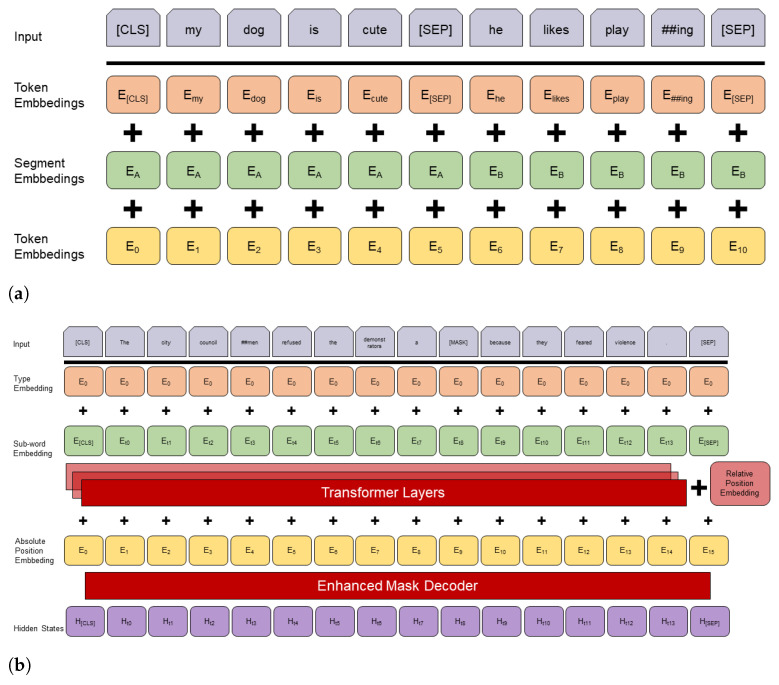
Comparison of the archictures of BERT and DeBERTa. (**a**) schematically depicts the architecture of BERT (modified from [13]). (**b**) shows in a similar way the architecture of DeBERTa (modified from [29]).

One significant factor for the performance of neural language models is the quality of their representations or embeddings. Refining the embedding can help the model to gain a more detailed understanding of the words. One way to achieve this is done by increasing the scope of the contextualized word embeddings. Ref. [13] was the first to implement a *deeply bidirectional* model based on contextualized and pre-trained representations with their bidirectional encoder representations from transformers (*BERT*). Technically, BERT is a transformer encoder stack. Its architecture is depicted in Figure 9a. It encodes an input into its representation. BERT uses many sophisticated concepts to produce representations. One major concept is bidirectionality. Bidirectionality means that both the next and previous words determine the representation of a word.

One example could be the word “bank”. The different meanings and thereby representations of it in the two sentences “I am going to the bank to sit down” and “I am going to the bank to withdraw cash” can only be understood by allowing the succeeding words “sit” and “withdraw” to influence the representation. Unidirectional models would only include the parts of the sentence which are previous to the word “bank”, in both cases, “I am going to the”. By considering both directions, the previous and succeeding neighbours, a more complete and differentiated representation of the word “bank” is possible.

BERT accomplishes the bidirectionality by using the masked language modelling (*MLM*) pre-training objective. MLM randomly selects a subset of tokens that either replaces some of the input tokens with the <MASK> token or with a random token or leaves them unchanged. This process is called masking. MLM aims to predict the original vocabulary of the masked token only on the basis of its context. After the prediction, the masked is replaced with the predicted token, except in cases where the masked token was not replaced in the first place. The embedding of the token enables the incorporation of information about the left and the right neighbouring tokens: in other words, both directions of its context. The idea behind not always replacing the tokens selected for masking with <MASK> aims to mitigate the mismatch between pre-training and fine-tuning since the <MASK> token does not appear during fine-tuning. Furthermore, there are other ways to achieve bidirectionality than MLM. XLNet (Generalized Auto-regressive Pre-training for Language Understanding) [27]. It uses permutations of each sentence to take the two contexts into account and uses two-stream self-attention to capture the tokens’ positional information.

Additionally, bidirectionality [13] includes another concept that significantly helped to improve BERT’s performance: next sentence prediction (*NSP*). It increases the model’s capability of understanding the relationship between two sentences. Many NLP tasks rely heavily on the understanding of these relationships. It is a binarized task, in which the model decides if a sentence is followed by another (label: IsNext) or not (label:NotNext).

### 4.7. Disentangled Bidirectional Representations

Ref. [8] presented a new model architecture, *DeBERTa* (decoding-enhanced BERT with disentangled attention), that builds on BERT [13] and RoBERTa [28] and improves their results by introducing two novel techniques: a disentangled attention mechanism and an enhanced masked decoder.

*Disentangled attention.* BERT represents each word by a single vector. This vector is computed by the sum of its word embedding and an absolute position embedding. DeBERTa separates or disentangles these embeddings into two different vectors, thereby representing each word by two vectors. The attention weights are computed using disentangled matrices on their word and *relative position*. The idea behind this approach is that the attention weight of a word pair not only depends on the content of the words but also on their relative position. Consider the words “deep” and “learning”. Their dependency is much stronger when their position to each other is smaller than when their position to each other is larger.

*Enhanced mask decoder* BERT uses masked language modelling (MLM). This is a fill-in-the-blank task, where the model learns to predict what word a token should mask, based on its context, i.e., the words surrounding the mask token. Ref. [8], p. 2, also uses MLM while pre-training. Their disentangled attention mechanism already considers the contents and relative positions of the context words but not their absolute positions. The absolute positions are in many cases crucial and can help the model learn syntactical nuances which would not be tangible if it only considered the relative position. Consider this example:

“a new store opened beside the new mall”
“a new <mask1> opened beside the new <mask2>”

**Figure 10:**
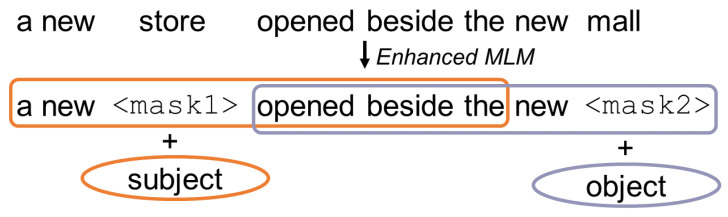
Illustration of processing an example with enhanced MLM.

The masked tokens “store” and “mall” have a similar context but store is the subject and mall is the object. This example is illustrated in Figure 10. These syntactical nuances can only be learned by considering the absolute position of the masked tokens. DeBERTa incorporates these absolute word embeddings before the Softmax layer, right before the model decodes the masked words based on the contextual embedding (word contents and positions). Thereby, ref. [8], p. 2, not only considers the findings of [25] but combines the relative and absolute position to exceed the results of previous models. Figure 9 illustrates the DeBERTa architecture and shows the way the BERT-like representation is supplemented by the relative position embedding and the enhanced mask decoder.

### 4.8. Token Replacement

**Figure 11:**
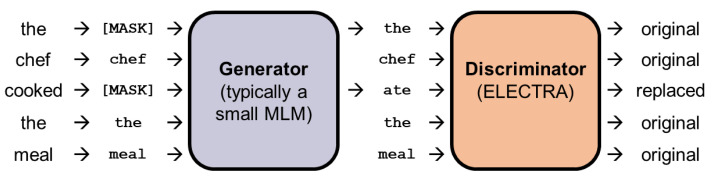
Illustration of the replaced token detection mechanism used in ELECTRA (modified from [12]).

BERT and DeBERTa [8] both use MLM. Ref. [12] proposed a new approach to further exploit the benefits of MLM. Their *ELECTRA* model consists of two encoders: the *generator* and the *discriminator*. Firstly, some of the input tokens are replaced with the [MASK] token. The generator then learns to predict the original identities of the masked-out tokens and replaces the masked-out tokens with its predictions. Then, the discriminator is trained to identify tokens that have been replaced by the generator’s tokens. Figure 11 depicts the process of masking, replacing, and discriminating tokens. Ref. [12] showed that this approach works well on relatively smaller computing resources and still outperforms previous models such as BERT.

### 4.9. Parameter Reduction

Approaches like DeBERTa [8] and other larger models explored the boundaries of how large a model can become thanks to modern hardware. They showed what a positive impact a large number of parameters and a big data set can have on the model performance. To empower teams and organizations with smaller resources, ref. [10] proposed their *ALBERT* (A lite BERT for self-supervised learning of language representations) in which they showed strategies for optimizing the efficiency of existing models. ALBERT optimizes the original BERT approach by two parameter reduction techniques:

*Factorized Embedding Parameterization:* ref. [10] separated the size of the hidden layers from the size of vocabulary embedding. This is done by decomposing the large vocabulary embedding matrix into two small matrices. Through this approach, it becomes easier to grow the hidden size without significantly increasing the parameter size of the vocabulary embeddings.

*Cross-Layer Parameter Sharing:* The idea of sharing parameters across layers has been previously explored with the transformer architecture [7]. However, ref. [10] focused more on the pre-training/fine-tuning setting, and prior works focused more on training for standard encoder-decoder tasks. Sharing parameters across layers prevents the parameter from growing with the depth of the network.

In addition to the parameter reduction techniques, ALBERT also includes an approach to exceed BERT in terms of sentence-level understanding. Refs. [28,30] discussed the ineffectiveness on next sentence prediction loss in BERT [13]. To overcome this weakness, ref. [10] proposed a self-supervised loss for sentence-order prediction (SOP), which primarily focuses on inter-sentence coherence. As a consequence of applying all these techniques, ALBERT outperforms the original BERT on well-known tasks such as GLUE while having considerably fewer parameters.

### 4.10. Multi-Task Learning

**Figure 12:**
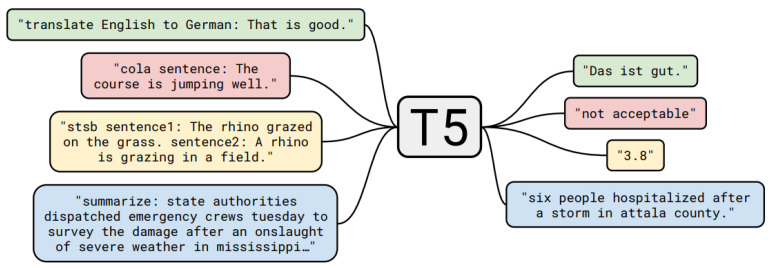
Example of inputs and outputs of T5 (Source: [11], (p. 3)).

Previously discussed models are based on the sequence-to-sequence framework. A certain input sequence leads to a certain output sequence produced by the model. The task that the model tries to solve is fixed and cannot be changed. By extending this framework into the *text-to-text framework* in their “Text-to-Text Transfer Transformer” (*T5*), the authors of [11] try to overcome this limitation of models that can only be applied to one fixed task. It is a transformer-based model using this text-to-text framework. The basic idea of the text-to-text framework is to treat every NLP problem as a “text-to-text” problem, i.e., text as input and new text as output. The input not only incorporates the data, which should be processed, but also information to indicate the specific task, thereby allowing for the model *multi-task learn*, because many different tasks can now be considered as one general “text-to-text” task. Figure 12 shows example inputs and outputs of T5’s text-to-text approach. There are several alternative approaches for multi-task learning with NLM. For instance, ML-DNN [31] uses task-specific layers that share an encoder layer stack.

Before applying any input, it has to be brought or unified into the text-to-text structure. One way of unifying the input is to transform every task into one static structure. Ref. [32] did this and formulated everything as a question-answer structure. Ref. [11], p. 11, did it differently: they selected a specific structure for each of their tasks. This allowed them to always train using maximum likelihood. Here are a few examples of how tasks are pre-processed before generating a corresponding output with T5:

**SQuAD:** question: <question> context: <context>
**CNN/Daily Mail:** summarize: <text>
**WMT English to German:** translate English to German: <text>
**MRPC:** mrpc sentence1: <sentence1> sentence2: <sentence2>

All these different tasks are mixed into one large fine-tuning data set. This is possible because of their common text-to-text task schema. The text-to-text framework allows [11], p. 2, to apply the same model, objective, training procedure, and decoding to every task, which can be formulated into a text-to-text structure. Therefore, T5 can be fine-tuned on a broad variety of English-based NLP tasks. Ref. [11], p. 7, used the “Colossal Clean Crawled Corpus” (or *C4* for short) for pre-training the model. This data set was also introduced in [11], p. 7, and is a lot larger (750GB) than most of the data sets used for NLP pre-training. In comparison, GPT was trained on the BookCorpus 4 GB [11], p. 26, and BERT on Wikipedia data and the book corpus combined 20 GB [11], p. 26. Since C4 is based on the common crawl project, it not only contains book-like or article-like texts but a much wider variety of English texts. Despite its colossal size [11], p. 7, ensured that, by their used techniques, C4 only consists of reasonably clean and natural English text.

Despite many of the top-performing transformer-based model architectures for NLP only consisting of a single “stack” (e.g., the encoder stack in BERT [13]), the authors in [11] have decided to use the standard encoder-decoder structure because their experiments indicated that it achieved good results on generative as well as classification tasks. The base model in [11], p. 7, is called T5-base. It consists of an encoder-decoder model. The encoder and the decoder are both similar in size to bert-base [11], p. 11, resulting in a model twice as large as bert-base because BERT only consists of an encoder. In addition to these novel features, T5 incorporates the techniques of the previously discussed ALBERT [10] (Section 4.9).

### 4.11. Use Case of Transformer Models

**Figure 13:**
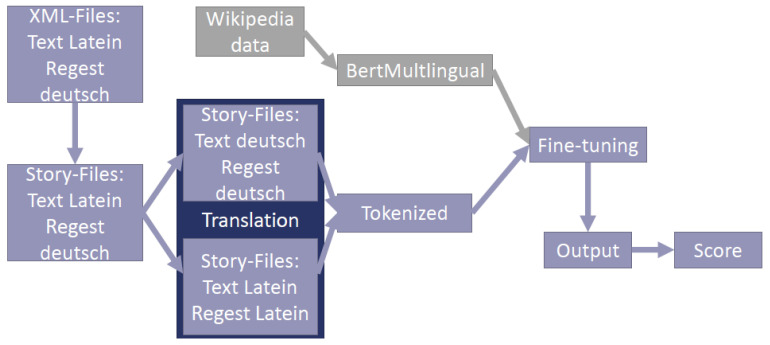
Illustration of the architecture used in [34].

There are several task-specific enhancements or variations of the previously discussed approaches and architectures. One specific example is BertSUM. It was introduced in [33]. It optimizes and modifies the original BERT architecture to achieve better results in extractive text summarization. We applied BertSUM in the field of medieval Latin documents [34]. By using a multi-lingual pre-trained BERT version and a translation service, we were able to generate German summaries of the Latin documents. Figure 13 illustrates the architecture of [34] and shows how summarization and translation can be combined.

### 4.12. Further Techniques

There are several interesting and promising techniques used in language representation models which are not directly linked to high-performing models in the GLUE leader board: for instance, the family of generative pre-trained transformer (GPT). It consists of generative models, which were trained unsupervised. The most recent are GPT-2 [35] and GPT-3 [36]. GPT-2 was trained to predict the next word in a large text corpus. GPT-3 further improved the concepts of GPT-2 and is currently the largest language model with 175 billion parameters. Even though it was not fine-tuned, GPT-3 could achieve similar performance to many state-of-the-art models when using few-shot learning. For example, [37], inspired by GPT-3, presented a suite called LM-BFF with simple and complementary techniques for fine-tuning language models on a small number of annotated examples, further substantially improving the performance.

Another mentionable model is BART [38]. It takes the idea behind MLM further by firstly corrupting the text with an arbitrary noising function and secondly training a model to de-noise (reconstruct) the original text.

## 5. Limitations

This work does not claim to cover all aspects of successful neural language models. It aimed at explaining, discussing, and contextualizing the most important aspect derived in the way described in Section 3 with a focus on the models’ performance as measured by the GLUE score. One threat to this approach is that it is biased towards models which are specialized and optimized to perform well under the measurement metrics of GLUE. The overview lacks the coverage of other models or solutions which might perform well in other specific tasks. Additionally, it lacks the coverage of other models not being submitted to be evaluated under GLUE score and hence not listed in GLUE leaderboard. Additionally, the coverage only includes the recent models developed since 2016. Older models might still have some room for improvements and could potentially be interesting for further research and development. Nevertheless, the GLUE benchmarking is used to objectively measure the models’ performance against a determined baseline. Including other models would be a difficult task since the comparisons would have to be based on some arbitrary parameters and not ones agreed upon by the community.

## 6. Discussion

The most recently published research on transfer learning and bidirectional models has by far outperformed previous approaches in different disciplines. Further development in these two fields could be very promising to even increase today’s state-of-the-art models. Future developments in the following areas are of special interest currently:

*Model efficiency*: Ref. [11], p. 42, showed with their experiments on the C4 data set and on the T5 model that larger models tend to perform better. The rapid development in making hardware faster and cheaper makes work with larger models possible. Transfer learning increases the performance of models on low-resource tasks (settings with a lack of assets to label more data). Thus, further research in developing smaller and cheaper models makes it possible to apply transfer learning where it has the most impact. Ref. [12], for instance, showed how increasing the complexity of a sub-task in a model (in their case, MLM) can lead to a decrease in the overall computing costs to develop a model. Ref. [10] showed that by optimizing the vocabulary and the parameter sharing, a model can significantly reduce its overall number of parameters while still achieving state-of-the-art results.

*Compositional Generalization*: Ref. [8], p. 10, marked an important milestone by surpassing human performance on SuperGLUE. Nonetheless, the machine learning models are not close to being capable of reaching human-level intelligence in natural language understanding (NLU). Humans are especially good at compositional generalization. This means the ability to leverage the knowledge learned from different tasks to solve novel compositions (new tasks) of familiar constituents (subtasks or basic problem-solving skills). Models which incorporate compositional structures in a more explicit manner, which could allow for combining neural and symbolic computation of natural language, could become even more closer to reaching human-level intelligence in NLU. These compositional structures could, for instance, be developed based on the progress in the field of multi-task learning, such as in [11].

*Measurement of Model Capabilities*: Due to the quick progress of neural language models, ways of how to measure the quality of the output of models are hitting their limitations. As discussed earlier, the fact that DeBERTa [8], p. 10, beat the human performance in the SuperGLUE task is remarkable. However, the NLU capability of the model is still far behind human performance. Most of the quality metrics such as ROUGE work by comparing the generated output to a gold standard. Thus, they always have a bias towards this gold standard and the similarity metric. One way of overcoming this bias is human-in-the-loop methods. Ref. [39] showed how to additionally fine-tune pre-trained models with direct human feedback. Their results beat models without this approach on classic metrics such as GLUE. They used online reinforcement learning in fine-tuning that lead to complex machine learning and software systems. They described their system as not easy to debug and that the quality of the human feedback was hard to control.

*Generalized Models*: With the rise of home assistants, models which can solve multiple tasks are in demand. These models should support users in various everyday life situations. The multi-task approach of [11], p. 42, showed very promising results. Their way of encoding multiple tasks into a text-to-text format seems to be well applicable to home assistants. Further research can be done in developing a wider variety of tasks. Another way of generalizing models is by increasing their capability of understanding multiple languages. Home assistants could expand to environments, where they are exposed to many languages, such as hotel lobbies or airport counters in the future. In those environments, their ability to solve multiple tasks in multiple languages could be a primary factor to determine their usefulness.

The rapid development in the field of neural language models especially in the last five years is indicating that future models could become better and better in performing in these rather difficult areas.

## Figures and Tables

**Table 1:** Comparison of the GLUE Score (* as reported by authors in the papers), the amount of steps and amount of parameters of the baseline version of the key models, which are further discussed in this paper.

Model	GLUE *	Steps	Parameters	Source
DeBERTa	90.8	1 M	134 M	[8,9], (p. 19)
ALBERT-base	89.4	1 M	12 M	[10], (pp. 5, 9)
T5-Base	85.97	786 K	220 M	[9,11], (pp. 11, 32)
ELECTRA-Base	82.7	766 K	110 M	[9,12], (pp. 6, 14)
Bert-base	78.3	1 M	108 M	[9,13]

## Data Availability

Publicly available datasets were used in this study. These data can be found here: https://gluebenchmark.com/ (last accessed on 2 January 2021).

## Abbreviations

The following abbreviations are used in this manuscript:

MLM	Masked Language Modelling
NLP	Natural Language Processing
NLU	Natural Language Understanding
NSP	Next Sentence Prediction
RNN	Recurrent Neural Networks
SOP	Sentence-Order Prediction
